# Pancreatic polypeptide and its central Y_4_ receptors are essential for cued fear extinction and permanent suppression of fear

**DOI:** 10.1111/bph.13456

**Published:** 2016-05-19

**Authors:** D Verma, B Hörmer, K Bellmann‐Sickert, V Thieme, A G Beck‐Sickinger, H Herzog, G Sperk, R O Tasan

**Affiliations:** ^1^ Department of PharmacologyMedical University InnsbruckInnsbruckAustria; ^2^ Institute of BiochemistryLeipzig UniversityLeipzigGermany; ^3^ Neuroscience Research ProgramGarvan Institute of Medical ResearchSydneyAustralia

## Abstract

**Background and purpose:**

Avoiding danger and finding food are closely related behaviours that are essential for surviving in a natural environment. Growing evidence supports an important role of gut‐brain peptides in modulating energy homeostasis and emotional‐affective behaviour. For instance, postprandial release of pancreatic polypeptide (PP) reduced food intake and altered stress‐induced motor activity and anxiety by activating central Y_4_ receptors.

**Experimental approach:**

We characterized [K^30^(PEG2)]hPP_2‐36_ as long‐acting Y_4_ receptor agonist and injected it peripherally into wildtype and Y_4_ receptor knockout (Y4KO) C57Bl/6NCrl mice to investigate the role of Y_4_ receptors in fear conditioning. Extinction and relapse after extinction was measured by spontaneous recovery and renewal.

**Key results:**

The Y4KO mice showed impaired cued and context fear extinction without affecting acquisition, consolidation or recall of fear. Correspondingly, peripheral injection of [K^30^(PEG2)]hPP_2‐36_ facilitated extinction learning upon fasting, an effect that was long‐lasting and generalized. Furthermore, peripherally applied [K^30^(PEG2)]hPP_2‐36_ before extinction inhibited the activation of orexin‐expressing neurons in the lateral hypothalamus in WT, but not in Y4KO mice.

**Conclusions and implications:**

Our findings suggests suppression of excessive arousal as a possible mechanism for the extinction‐promoting effect of central Y_4_ receptors and provide strong evidence that fear extinction requires integration of vegetative stimuli with cortical and subcortical information, a process crucially depending on Y_4_ receptors. Importantly, in the lateral hypothalamus two peptide systems, PP and orexin, interact to generate an emotional response adapted to the current homeostatic state. Detailed investigations of feeding‐relevant genes may thus deliver multiple intervention points for treating anxiety‐related disorders.

**Abbreviations**
CS: conditioned stimulus
CS+: conditioned stimulus that was paired with an unconditioned stimulus
CS−: conditioned stimulus that was not paired with an unconditioned stimulus
NPY: neuropeptide Y
NTS: nucleus tractus solitarii
PP: pancreatic polypeptide
US: unconditioned stimulus

## Tables of Links

**Table:** 

**TARGETS**
**GPCRs**
Orexin‐1 receptors
Y_4_ receptors

**LIGANDS**
NPY, neuropeptide Y
Orexin
PP, pancreatic polypeptide

These Tables list key protein targets and ligands in this article which are hyperlinked to corresponding entries in http://www.guidetopharmacology.org, the common portal for data from the IUPHAR/BPS Guide to PHARMACOLOGY (Pawson *et al*., 2014) and are permanently archived in the Concise Guide to PHARMACOLOGY 2015/16 (Alexander *et al*., 2015).

## Introduction

Avoiding danger and finding food are two intimately associated, life‐sustaining behaviours that are strongly modulated by emotions (Sakurai and Mieda, 2011). Maladaptation within such survival circuits can induce dysregulated, pathological behaviour, resulting in the development of feeding or anxiety disorders (Myers and Davis, 2007; Finger *et al.*, 2010; Sternson, 2013; Sternson *et al.*, 2013). In the USA and Europe, an estimated number of 100 million people are suffering from anxiety‐related diseases (Wittchen *et al.*, 2011; Kessler *et al.*, 2012). Thus, identifying critical components of the underlying circuitry may provide new treatment strategies. Interestingly, neuropeptides are essential modulators of both energy homeostasis and anxiety‐related behaviours (Hokfelt *et al.*, 2003; Coll *et al.*, 2007; Bowers *et al.*, 2012; Schellekens *et al.*, 2012). For instance, gut–brain peptides, such as neuropeptide Y (NPY), are released during states of hunger or acute danger (Bowers *et al.*, 2012; Holzer *et al.*, 2012). While the anxiolytic and fear‐reducing properties of NPY are increasingly evident (Heilig, 1995; Karlsson *et al.*, 2008; Tasan *et al.*, 2011; Verma *et al.*, 2012), the role of pancreatic polypeptide (PP) and its Y_4_ receptor in fear‐related behaviour is still poorly understood.

PP, which preferentially activates Y_4_ receptors, is synthesized mainly in endocrine cells of the pancreas and released into the blood circulation by a cholinergic, vagus‐dependent mechanism predominantly regulating gastrointestinal processes and appetite (Lin *et al.*, 2009; Holzer *et al.*, 2012; Shi *et al.*, 2013). In the CNS, Y_4_ receptors have a very distinct distribution, with particular high expression in the hypothalamus and the nucleus tractus solitarii (NTS), two brain areas that are accessible to peripherally released PP and important for mediating emotionally driven autonomic responses (Dumont *et al.*, 2007; Tasan *et al.*, 2009). Thus, PP may be considered as a signalling peptide communicating the internal metabolic state to respective CNS centres that generate an adaptive behavioural response. This may include satiety signals but also accompanying emotional behaviour, such as altered anxiety and fear processing.

Recently, we demonstrated that genetic deletion of Y_4_ receptors (Y4KO) results in increased novelty‐induced motor activity, reduced anxiety and improved stress coping (Painsipp *et al.*, 2008; Tasan *et al.*, 2009; Painsipp *et al.*, 2010). Overexpression of the endogenous Y_4_ receptor agonist PP, on the other hand, resulted in reduced anxiety‐like behaviours. Interestingly, chronic peripheral application of PP reduced anxiety‐like behaviours, while i.c.v. injection of PP had no effect (Asakawa *et al.*, 1999; Asakawa *et al.*, 2003), suggesting that only peripherally released PP may properly interact with central Y_4_ receptors.

Fear and anxiety‐related behaviours are predominantly controlled by the amygdala complex and can be tested by Pavlovian fear conditioning, a simple form of associative learning (LeDoux, 2000). In the fear conditioning paradigm, an unconditioned stimulus (US), usually a mild electric foot shock, is repetitively paired with a conditioned stimulus (CS), typically represented by an auditory stimulus. After a few pairings, the CS alone can elicit a typical fear reaction, termed conditioned response (CR). Subsequently, repetitive presentations of the CS in the absence of the US result in a gradual decrease of the learned fear response, a process called fear extinction. Recent evidence suggests that central NPY inhibits the expression and promotes the extinction of *learned fear* (Gutman *et al.*, 2008; Fendt *et al.*, 2009; Verma *et al.*, 2012). Furthermore, we demonstrated that short‐term fasting impairs fear consolidation but facilitates fear extinction by increasing feedforward inhibition in an amygdala microcircuit (Verma *et al.*, 2016). This feedforward inhibition was reduced in Y4KO mice resulting in impaired fear extinction. These data indicate an important role of gut‐derived peptides in emotional control, thus linking life‐sustaining circuitries for fear and hunger.

Here, we have characterized the role of peripheral PP in acquisition, recall/expression and extinction of conditioned fear and investigated the particular contribution of Y_4_ receptors in these processes. We decided to stimulate Y_4_ receptors by peripheral injection of a novel derivative of PP, the PEGylated peptide [K^30^(PEG2)]hPP_2‐36_, which we have characterised as a selective agonist of Y_4_ receptors . While both lipidation and PEGylation of PP prolong plasma half‐life, PEGylation also inhibits arrestin recruitment and receptor internalization, two effects that are independent of the PEGylated amino acid residue (Mäde *et al*., 2014) and may significantly prolong the activation of Y_4_ receptors. Our results showed that [K^30^(PEG2)]hPP_2‐36_ acting on central Y_4_ receptors promoted the cued fear extinction probably by inhibiting orexin‐expressing neurons in the lateral hypothalamus, resulting in a stable, long‐term suppression of fear.

## Methods

### Synthesis and characterization of [K^30^(PEG2)]hPP_2‐36_

#### Peptide synthesis

The peptide was synthesized as described previously (Bellmann‐Sickert *et al.*, 2011) using fluorenylmethoxycarbonyl‐/tert‐butyl (Fmoc/tBu) protecting group strategy on a Rink amide resin (Iris Biotech, Marktredwitz, Germany; loading capacity: 0.7 mmol·g^−1^, mesh size 100–200). The peptide sequence was elongated automatically by a fully automated parallel peptide synthesizer (Syro II, Biotage, Uppsala, Sweden). Amino acids, N,N‐diisopropylcarbodiimide (DIC) and oxyma were dissolved in N,N‐dimethylformamide (DMF) to a final concentration of 0.5 M and applied with an 8‐fold excess. Double couplings of 2 × 30 min were performed per cycle. Fmoc deprotection was carried out with 40% piperidine in DMF for 3 min followed by 20% piperidine for 10 min. Lysine at position 30 was introduced with (4‐methoxyphenyl)diphenylmethyl (Mmt) side chain protection. N‐terminal proline was either introduced as *tert*‐butoxycarbonyl (Boc) protected or was labelled with 5‐carboxytetramethylrhodamine (TAMRA) using 2 equivalents TAMRA, 2 equivalents O‐(7‐azabenzotriazol‐1‐yl)‐N,N,N′,N′‐tetramethyluronium‐hexafluorophosphate (HATU) and 1.9 equivalents N,N‐diisopropylethylamine dissolved to 0.5 M in DMF and incubation for 2 h at room temperature. After complete synthesis of the peptide backbone, the Mmt group was cleaved off by repeatedly (15 times) applying 1 mL of a solution of 2% (v/v) trifluoroacetic acid (TFA) and 5% (v/v) triisopropylsilane (TIS) in dichloromethane for 2 min and washing with dichloromethane. For PEGylation, 2 equivalents of α‐methoxy‐ω‐carboxylic acid succinimidyl ester poly(ethylene glycol) (MeO‐PEG‐NHS, 2000 Da; Iris Biotech) were dissolved in DMF/dichloromethane (2:1 (v/v)) to a concentration of 0.06 M. 4‐dimethylpiperidine and DIC were added to a concentration of 0.1 M. The mixture was added to the resin and shaken for 16 h at room temperature and washed subsequently with DMF and dichloromethane. Peptide was cleaved from the resin with a mixture of TFA, 1,2‐ethanedithiol and *para*‐thioanisole (18:1:1, v/v/v) for 3 h at room temperature and precipitated from ice‐cold diethylether. Methionines were reduced by applying a mixture of TFA, trimethylsilylbromide and 1,2‐ethanedithiol (100:1.6:1.2, v/v/v) for 20 min at room temperature and precipitation from ice‐cold diethylether. Precipitates were washed with ice‐cold diethylether, dried under vacuum and dissolved in a mixture of water and *tert*‐butanol (3:1, v/v). Products were purified and analysed by reversed phase high performance liquid chromatography using a gradient consisting of 0.1% TFA in water (eluent A) and 0.08% TFA in acetonitrile (eluent B), ranging from 20% to 60% B in A for 40 min. Purification was done by using a Jupiter Proteo 90 Å with 10 μm pore size and dimensions of 21.2 × 250 mm (Phenomenex). Analytics were performed on a Jupiter Proteo 90 Å with 10 μm pore size and dimensions of 4.6 × 250 mm (Phenomenex, Aschaffenburg, Germany). Peptide purity was confirmed to be >95%. Peptide identity was proven by matrix‐assisted laser desorption/ionization mass spectrometry using an Ultraflex III matrix‐assisted laser desorption/ionization time‐of‐flight/time‐of‐flight (MALDI‐TOF/TOF) mass spectrometer (Bruker, Bremen, Germany). The MS results are shown in Supporting Information Figure S1.

#### Characterisation of activity at Y receptors

Agonist activity at Y receptors was assessed by measuring inositol phosphate accumulation in COS‐7 cells, as described previously (Hofmann *et al.*, 2013). We used COS‐7 cells that stably expressed one of the four human Y receptors (hYR) and a chimeric G_i/q_ protein (Kostenis *et al.,* 1997) to channel cAMP inhibition to phospholipase C stimulation and hence inositol 1,4,5‐triphosphate (IP_3_) production. Stably transfected COS‐7‐hYR‐GαΔ6qi4myr cells were cultivated in DMEM with 4.5 g·L^−1^ glucose and L‐glutamine supplemented with 10% (v/v) heat‐inactivated FCS, 100 units mL^−1^ penicillin, 100 μg mL^−1^ streptomycin, 1.5 mg mL^−1^ G418‐sulfate and 146 μg mL^−1^ hygromycin B in a humidified atmosphere at 37°C and 5% CO_2_. COS 7 cells were a gift from Prof. Dr. Torsten Schöneberg (Leipzig University). Stable cell lines were prepared as described in Mäde *et al*., 2014. Cells were grown in 48‐well plates (90 000 cells in 500 μL per well) for 24 h. Cells were labelled with 2 μCi mL^−1^ myo‐[2‐3H]‐inositol (PerkinElmer, Rodgau, Germany) in culture medium without penicillin and streptomycin for at least 16 h followed by stimulation for 1 h with peptide solutions (10^−5^–10^−11^ M, depending on ligand potency) in DMEM with 4.5 g L^−1^ glucose and L‐glutamine containing 10 mM LiCl (Sigma‐Aldrich). Cells were lysed applying 0.1 M sodium hydroxide for 5 min at room temperature followed by neutralization with 0.2 M formic acid. Samples were diluted in dilution buffer (5 mM Na‐borate and 0.5 mM Na‐EDTA). Cell debris was removed. Samples were loaded on columns packed with the anion exchange resin AG 1‐X8 formate (BIO‐RAD, Munich, Germany), washed with glycerolphosphate elution buffer (5 mM Na‐borate, 60 mM Na‐formate) and water and eluted with elution buffer (1.0 M ammonium formate, 0.1 M formic acid). Eluates were diluted with scintillation cocktail Ultima Gold (Perkin Elmer) and measured by a scintillation counter (Tri‐Carb 2910 TR from PerkinElmer). The values for decay per minute obtained at the highest used wild type concentration (corresponds to 100% activity) were normalized to a mean value to which all other agonists were related. For each compound, global mean EC_50_ and pEC_50_ ± SEM (*n* ≥ 2) were calculated from the entire normalized concentration‐response curves by nonlinear regression using GraphPad Prism 5.0. All compounds were examined in duplicate, in at least two independent experiments.

#### Metabolic stability in human blood plasma

Proteolytic stability in human blood plasma was assessed as described previously (Mäde *et al.*, 2014) with minor adaptations. 15 nmol of the N‐terminally TAMRA‐labelled compound were dissolved in 10 μL of distilled water and 1490 μL of human blood plasma (citrate stabilized). The mixture was incubated for 96 h at 37°C. At the indicated time points, samples (150 μL ) were withdrawn and equal volumes of ethanol and acetonitrile were added in order to precipitate plasma proteins. Samples were incubated at −20°C overnight and centrifuged at 12,000 g for 30 s. Supernatants were again incubated at −20°C for 20 min and afterwards filtered using SpinX tubes (0.22 μm, Costar). Filtrates were directly subjected to reverse phase high performance liquid chromatography (RP‐HPLC) using a Varitide RPC column (200 Å, 6 μm pore size, 4.6 × 250 mm) and a gradient of 20 to 60 % B in A for 30 min. Intact peptide was followed by fluorescence detection at an excitation wavelength of 525 nm and an emission wavelength of 572 nm. Degradation of each compound was independently performed twice (*n* = 2) and is presented as mean ± SEM (Supporting Information Figure S3)

### Animals

All animal care and experimental procedures were conducted in accordance with international laws and policies (Directive 2010/63/EU of the European parliament and of the council of 22 September 2010 on the protection of animals used for scientific purposes; Guide for the Care and Use of Laboratory Animals, US National Research Council, 2011) and were approved by the Austrian Ministry of Science. Animal studies are reported in compliance with the ARRIVE guidelines (Kilkenny *et al*., 2010; McGrath & Lilley, 2015). All effort was taken to minimize the number of animals used and their suffering.

Experiments were performed in adult male mice (10–16 weeks old, weighing 25–30 g). All mice were kept and bred in the animal facility of the Medical University of Innsbruck. Y4KO mice were backcrossed to a C57Bl/6NCrl background for at least 10 generations and compared with respective WT controls before the experiments. For both mouse lines, experimental animals were derived from homozygote breeding pairs. Generation of Y4KO mice has been described in detail previously (Sainsbury *et al.*, 2002). Deletion of Y_4_ receptor genes was confirmed in all mice used for the experiments by PCR and agarose gel electrophoresis. Further characterization of receptor deletion was performed in randomly selected mice by *in situ* hybridization and receptor autoradiography (using human [^125^I]‐PP as ligands for Y_4_ receptors), as described in detail previously (Gobbi *et al.*, 1998; Tasan *et al.*, 2009).

Mice were housed in groups of three to five animals per cage under standard laboratory conditions (12 h/12 h light/dark cycle, lights being on at 07:00 h, food and water *ad libitum*).

The mouse was chosen as an appropriate animal species, because we wanted to study the underlying cause and potential treatment options for human fear‐related disorders. Pavlovian fear conditioning was selected as a well‐established model to study the development and extinction of fear. Because human anxiety disorders can be only studied adequately in mammals, our results have no implications for replacement, refinement or reduction. However, the parameters of the behavioural experiments, including fear conditioning (Verma *et al.*, 2012) and fasting (Verma *et al.*, 2016), were refined according to our previous experiments and were selected as the least harmful possible while still allowing the study to be conducted.

#### Experimental conditions

**Figure 1:**
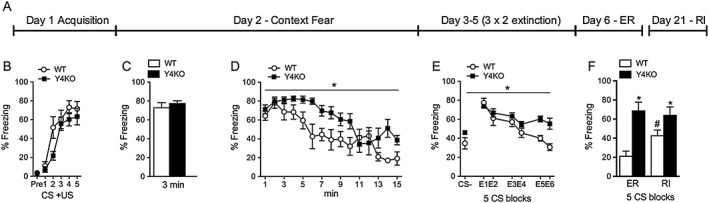
Context and cued fear extinction is impaired in Y4KO mice. (A) Experimental timescale of fear conditioning and extinction experiments; (B) compared with WT mice, Y4KO mice exhibit unchanged acquisition and (C) expression of context fear, but (D) delayed extinction of context fear and (E) impaired extinction of cued fear. (F) While WT mice display an increase in fear expression upon reinstatement (RI), Y4KO mice display equal freezing levels during extinction recall (ER) and reinstatement, suggesting complete absence of fear extinction memory (WT: *n* = 9, Y4KO: *n* = 8; data are expressed as mean ± SEM, *P < 0.05, significantly different from WT; # *P*<0.05, significantly different from ER).

The number of animals per experiment was based on previous data (Verma *et al.*, 2012) and a corresponding power analysis. The exact group size for the individual experiments is shown in the corresponding figure legends. In general, a group size of 7–10 animals was used. An unequal group size in Figures 1 and S2 was due to a limited availability of male Y4KO mice and respective, age‐matched controls.

For randomization, half the litter of wildtype (WT) mice was in the control group, while the other half was in the treatment group. Blinding was implemented as follows. The operator was blinded to the group identity, but not to animals of the same group. Thus, for i.p. injection of a drug, two different solutions were prepared: solution A (e.g. saline) and solution B (e.g. [K^30^(PEG2)]hPP_2‐36_). Importantly, the data analyst was blinded (histology), or analyses were performed by automated computer software (behavioural experiments).

Mice were killed by an i.p. injection of a lethal dose of thiopental (500 mg·kg^−1^; Sandoz, Austria) to remove tissue for histological analyses or by CO_2_ inhalation after the final behavioural experiments.

#### Behavioural experiments

For behavioural experiments, [K^30^(PEG2)]hPP_2‐36_ was dissolved in 0.9% saline to yield a final drug concentration of 1 mg kg^−1^.

##### Fear conditioning paradigm

Naïve WT (C57Bl/6NCrl) or Y4KO mice were used for fear conditioning experiments. Fear conditioning experiments were conducted during the light phase of the light/dark cycle in sound proof chambers (TSE Systems, Bad Homburg, Germany) by repetitive exposure of the mice to an auditory stimulus co‐terminating with a foot shock, as described in detail previously (Verma *et al.*, 2012). Fear conditioning and context testing were performed in *context A* consisting of a transparent acrylic mouse conditioning chamber with a metal grid floor. The whole set‐up was enclosed by a sound attenuating chamber. Illumination was 80 lux, and chambers were cleaned with 70% ethanol. Fear recall as well as fear extinction and extinction recall was performed in a different context consisting of a dimly illuminated (10 lux) chamber with black, smooth walls and the floor cleaned with 1% acetic acid (*context B*).

#### Fear conditioning procedure

On day 1 (context A), mice were subjected to a differential fear conditioning paradigm in which one auditory stimulus served as a CS because it was explicitly paired (CS+, 30 s white noise, 80 dB) with the US, whereas the second auditory stimulus was not paired (CS−, 30 s, 3.5 kHz, 80 dB). All animals received five CS− and five CS+ in an alternating order, starting with a CS+. The US co‐terminating with each CS+ consisted of a foot shock. The shock intensity was set to 0.5 mA (2 s), a minimum threshold at which both WT and Y4KO mice displayed respective behavioural reactions in the sensitivity analysis (Verma *et al.*, 2016). On days 2 and 3, mice were tested for their context (context A for 15 min) and CS‐related fear memory (context B) respectively.

#### Fear extinction procedure

Because male Y4KO mice on C57BL/6NCrl background did not show fear extinction, we performed an extensive extinction protocol. In these experiments, we performed a total of six extinction sessions (context B), each consisting of 15 CS+ (30 s, inter stimulus interval 5 s), and extinction recall was tested the following day (five CS+ presentations, context B). Reinstatement was induced by an unsignalled foot shock in context A (day 20) and tested on day 21 by exposing the mice to five CS+ in context B. Behaviour was recorded by a video camera and scored offline by a pixel‐based analysis software (http://topowatch.sourceforge.net/, TopoWatch v0.3). The parameters of the programme were validated by comparison with the manual analysis of two experienced observers. A detailed validation procedure of the analysis software for detecting freezing behaviour including baseline activity and reactive motor activity in an unfamiliar environment relevant for fear conditioning (e.g. the fear conditioning box) has been published previously (Verma *et al.*, 2012).

##### Peripheral injection of Y_4_ receptor agonist on fear‐related behaviour

Fear conditioning was performed in WT (C57Bl6/NCrl) and Y4KO mice on day 1 (context A) as described in the previous paragraph. To avoid interference with postprandial released PP, mice were fasted overnight (16 h) before injection of the drug followed by fear extinction training. Water was freely available during the fasting period. Based on our previous results, a 16 h fasting period was required to reach our experimental aim (Verma *et al.*, 2016). A control experiment was performed without food restriction (non‐fasting). [K^30^(PEG2)]hPP_2‐36_ (1 mg·kg^−1^) or saline was injected i.p. 60 min before behavioural testing, as indicated in the timeline of the respective experiments. For extinction experiments, mice were divided into two groups, receiving an i.p. injection of either the long‐acting Y_4_ receptor agonist [K^30^(PEG2)]hPP_2‐36_ (1 mg·kg^−1^) or saline in controls followed after 60 min by extinction training (extinction 1; 5 CS− and 25 CS+, context B). Mice were then tested on day 3 for extinction recall under drug‐free conditions by exposure to five CS+ (context B). To exclude any bias of group selection, another extinction session was performed on day 4 (extinction 2; 25 CS+, context B) followed by extinction recall on day 5 (five CS+; context B), both under drug‐free conditions. *Spontaneous recovery* (day 12) and *renewal* (day 13) were tested by exposing the mice to five CS+ in contexts B and A respectively.

##### Determination of motor activity after [K^30^(PEG2)]hPP_2‐36_ injection

Male WT mice were injected i.p. with either the long‐acting Y_4_ receptor agonist [K^30^(PEG2)]hPP_2‐36_ (1mg kg^−1^) or saline. Activity measurements were performed for 24 h in a novel cage. Mice were single‐housed in standard cages with food and water *ad libitum*. Movements of the mice were detected by an infrared sensor throughout light and dark phase (TSE LabMaster InfraMot, Bad Homburg, Germany).

### Histochemical analysis

A separate group of WT and Y4KO mice were fasted overnight (16 h), injected i.p. with [K^30^(PEG2)]hPP_2‐36_ or saline and subjected to extinction training 60 min later. Using a cryostat 40 μm coronal, free‐floating sections were cut for subsequent immunohistochemistry.

#### Immunohistochemistry procedures

Immunohistochemical analysis was performed on free‐floating, PFA‐fixed, 40 μm thick coronal sections using indirect peroxidase labelling, as described previously (Tasan *et al*., 2011). The following antisera were used: polyclonal rabbit anti‐c‐Fos (1:20 000 PC38, Calbiochem) and polyclonal rabbit anti‐pan‐orexin (1:1000, Abcam ab6214, Lot:GR63996‐1). In brief, coronal sections were incubated free‐floating in 10% normal goat serum (Biomedica, Vienna, Austria) in Tris–HCl buffered saline (TBS; 50 mM, pH 7.2) for 90 min, followed by incubation with primary antiserum. The resulting complex was visualized by incubation with HRP‐coupled secondary antibody (1:250 P0448; Dako, Vienna, Austria) at room temperature for 150 min, followed by tyramide amplification solution (1:100, TSA Fluorescein) for 6 min. After staining, sections were exposed to 0.01 M of HCl for 20 min at room temperature to denature HRP and first primary antibodies, and incubation with the second primary antibody was performed as described for c‐Fos except that TSA CY3 was used for staining. Sections were mounted on slides and covered using Vectashield mounting medium (Vector laboratories, Inc., Burlingame, CA, USA).

The number of c‐Fos positive cells was obtained bilaterally from six matched sections per animal showing the lateral hypothalamus, at a magnification of 400×, in multiple separate fields and mean values were calculated for each mouse. Results are presented as number of immunoreactivity positive cells per section and expressed as mean ± SEM. Analysis of dual labelling immunofluorescence was carried out as described elsewhere (Tasan *et al*., 2011). In brief, for each brain area containing a region of interest, four matched sections per mouse (seven mice per group) were processed for either c‐Fos/orexin for dual localization. Identification of dual‐labelled cells was performed at 400 times magnification within the respective brain area in each section.

#### Data and statistical analysis

These studies comply with the recommendations on experimental design and analysis in pharmacology (Curtis *et al*., 2015). Results are presented as means ± SEM. No data normalization was employed. Data were analysed for normal distribution and equal variances using GraphPad Prism software (Prism 5 for Macintosh; GraphPad Software Inc., San Diego, CA, USA). All acquisition and extinction experiments were analysed by repeated two‐way ANOVA for time, genotype/treatment and interaction. *P* < 0.05 was considered as statistically significant. If F achieved *P* < 0.05 and no variance inhomogeneity was observed, a Bonferroni *post hoc* test was performed for selected comparisons. Comparisons involving two groups were analysed by Student's t‐test or by the Mann–Whitney test for non‐parametric values.

## Results

### Pharmacological characterization of [K^30^(PEG2)]hPP_2‐36_

#### Agonist activity of the peptide at human Y receptors

Activity at the human receptors, hY_1_, hY_2_, hY_4_ and hY_5_, was measured by accumulation of IP_3_, after channeling cAMP inhibition to phospholipase C activity, in COS7 cells. Results from the concentration‐response studies are shown in Supporting Information Figure S2 and summarized in Table 1. This modified PP peptide, [K^30^(PEG2)]hPP_2‐36_, was found to selectively activate hY_4_ receptors with an EC_50_ of 9.1 nM, whereas with all other Y receptors, no full curves could be obtained over the concentration range used (10^−11^ –10^‐5^M). For comparison, the native human PP has an EC_50_ value of 1.3nM at the hY_4_ receptor (Bellmann‐Sickert *et al.*, 2011; Mäde *et al.*, 2014), indicating that PEGylation had only a minor effect on agonist potency.

**Table 1:** Agonist activity of the peptide [K^30^(PEG2)]hPP_2‐36_ at human Y receptors.

hY_1_		hY_2_		hY_4_		hY_5_	
EC_50_	pEC_50_	EC_50_	pEC_50_	EC_50_	pEC_50_	EC_50_	pEC_50_
> 1000	n. d.	> 1000	n. d.	9.1	8.0 ± 0.2	402	6.4 ± 0.1

Peptide activity determined from concentration‐response curves from the inositol phosphate turnover assay. Inositol phosphate accumulation was measured by stimulating COS7 cells stably transfected with the respective Y receptor subtype and a chimeric G_i/q_ protein, over a range of peptide concentrations (Supporting Information Figure S2). Experiments were performed in duplicate, at least twice. Data in the Table are EC_50_ (shown as nM) and pEC_50_ (± SEM) values, generated by nonlinear regression using GraphPad Prism 5.0. n.d., not determined.

#### Proteolytic stability assay in human blood plasma

Peptide stability was evaluated by incubating a fluorescently labelled derivative in human blood plasma and subsequent chromatographic analysis of fluorescent degradation products. TAMRA‐[K^30^(PEG2)]hPP_2‐36_ exhibited a high degree of stability towards proteolysis with 92% intact peptide after an incubation period of 96 h (Supporting Information Figure S3). This is in accordance with previous results for different hPP analogues that all showed a substantial elevation of proteolytic stability in different media. The native hPP showed about 60% intact compound after incubation for 96 h, under the same experimental conditions (Bellmann‐Sickert *et al.*, 2011; Mäde *et al.*, 2014).

### Behavioural experiments

#### Fear conditioning and extinction in Y4KO mice

In order to investigate the role of Y_4_ receptors in learned fear, we subjected Y4KO mice to a fear conditioning and extinction paradigm. As shown previously (Verma *et al.*, 2016), sensitivity to the US, baseline freezing and acquisition of conditioned fear was similar in WT and Y4KO mice (Figure 1B). Context fear and context extinction was tested 24 h after fear conditioning by analysing freezing behaviour in context A for 3 min and 15 min respectively. Recall of context fear was unchanged (Figure 1C); however, compared with WT mice, extinction of context fear was significantly delayed in Y4KO mice (Figure 1D, F_(1/12)_ = 5.46, *P* < 0.05).

Cued fear extinction was determined by the change of the freezing response to the CS on six individual extinction sessions, each consisting of 15 CS presentations (Figure 1A and E; blocks of the first five CS are depicted for each session). In contrast to context extinction that was intact but delayed, cued extinction learning was largely impaired (Figure 1E, F_(1/13)_ = 6.58, *P* < 0.05). To test if Y4KO mice exhibit residual CS+ related fear extinction, we tested for stress‐induced re‐emergence of fear by subjecting the mice to an unsignalled foot shock in context A followed by reinstatement testing in context B on day 20 and 21 respectively. While WT mice exhibited robust reinstatement (*t* = 2.4, *P* < 0.05), freezing levels in Y4KO mice were equally high during CS test (day 3) and extinction recall (day 6) and also during reinstatement testing on day 21, suggesting complete absence of fear extinction memory.

### Role of peripheral PP in the modulation of motor activity

PP is the main ligand for the Y_4_ receptor. It is produced in the pancreas and released upon ingestion of food. Thus, to investigate the effect of peripherally released PP and its relation to Y_4_ receptors, we injected the long‐acting Y_4_ receptor agonist [K^30^(PEG2)]hPP_2‐36_ i.p. into WT and Y4KO mice, followed by behavioural testing.

To exclude non‐specific effects of [K^30^(PEG2)]hPP_2‐36_ on motor activity that may compromise freezing detection during extinction training, we first injected male WT mice i.p. with [K^30^(PEG2)]hPP_2‐36_ or saline and subsequently observed the activity of the mice for 24 h in a novel cage (Supporting Information Figure S4). Repeated two‐way ANOVA revealed no difference between treatment groups (F_(1/13)_ = 0.03, *P* > 0.05), but different activity over time (F_(23/299)_ = 8.93, *P* < 0.05), indicating that i.p. injection of [K^30^(PEG2)]hPP_2‐36_ did not influence general motor activity and preserved physiological activity changes during light/dark cycles.

### Effect of peripherally injected [K^30^(PEG2)]hPP_2‐36_ on cued fear extinction

**Figure 2:**
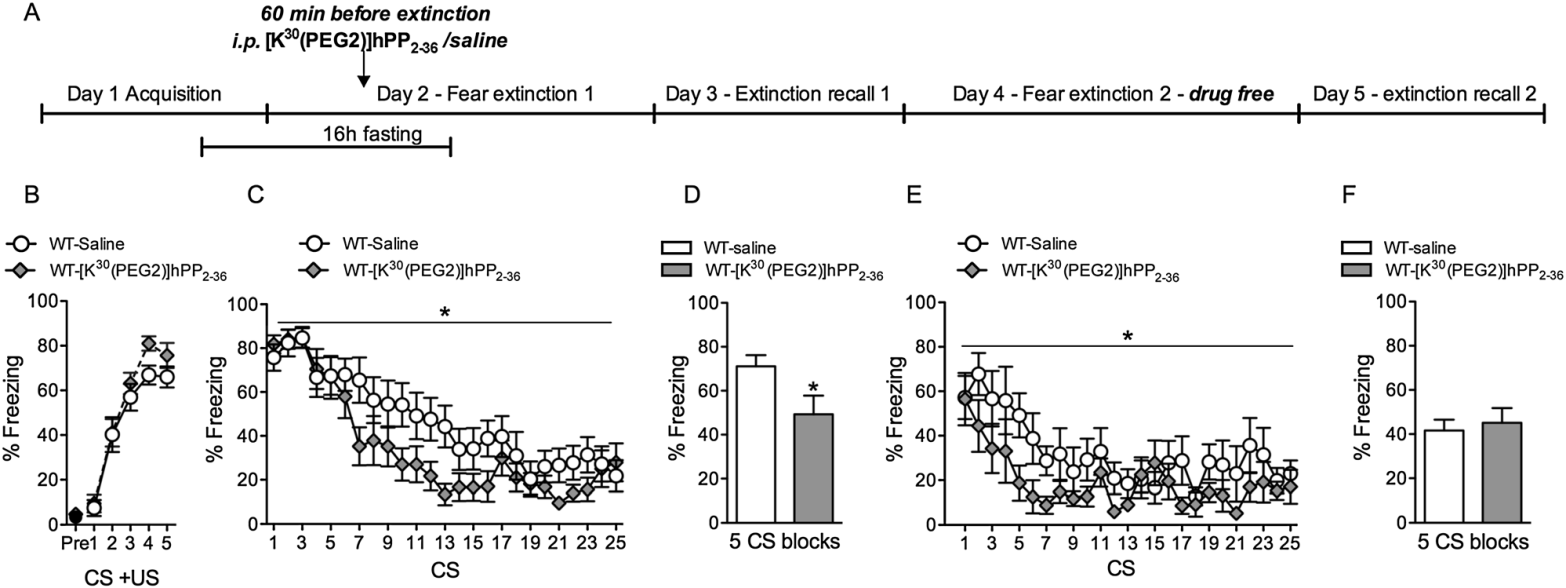
Cued fear extinction is facilitated by peripheral injection of the long‐acting Y_4_ receptor agonist [K^30^(PEG2)]hPP_2‐36_. (A) Experimental procedure of fear conditioning and extinction experiments. (B) Following fear acquisition, male WT mice were divided into two equal groups; (C) peripheral injection of [K^30^(PEG2)]hPP_2‐36_ in fasted mice (16 h, before and during fear extinction) resulted in facilitated extinction of CS‐induced fear compared with saline‐injected controls. (D) Freezing to the CS was reduced in the [K^30^(PEG2)]hPP_2‐36_ group during extinction recall tested under drug‐free conditions with food available *ad libitum*. (E) An additional fear extinction session of the saline and [K^30^(PEG2)]hPP_2‐36_ groups under drug‐free and fed conditions resulted in (F) equal freezing levels during extinction recall 2, demonstrating similar composition of the groups (saline: *n* = 8, [K^30^(PEG2)]hPP_2‐36_: *n* = 8) Data are expressed as mean ± SEM, *P < 0.05, significantly different from saline.

Specific impairment of cued fear extinction in Y4KO mice suggests that activation of Y_4_ receptors may facilitate fear extinction. Thus, to investigate if peripherally released PP is the main actor mediating the effect of Y_4_ receptors on cued fear extinction learning and to differentiate acute effects of Y_4_ receptor stimulation from permanent Y_4_ receptor deletion, we injected [K^30^(PEG2)]hPP_2‐36_ i.p. into male WT mice 60 min before fear testing and consecutive extinction training (Figure 2A). Following fear acquisition, male WT mice were divided into two equal groups (Figure 2B). All mice were fasted overnight for 16 h to avoid interference with endogenous PP release that naturally occurs after feeding. As shown in Figure 2C, i.p. injection of [K^30^(PEG2)]hPP_2‐36_ significantly facilitated extinction learning in WT mice (F_(1/22)_ = 5.80, *P* < 0.05). The effect of facilitated fear extinction was consolidated into an improved extinction memory as reflected by a reduction of freezing during extinction recall performed 24 h later under drug‐free and non‐fasting conditions (Figure 2D, t_(14)_ = 2.20, *P* < 0.05). A consecutive extinction trial (Figure 2E, extinction 2) was performed on day 4 followed by extinction recall 2 on day 5, both in the absence of drug and with food and water available *ad libitum*, demonstrating that extinction under drug‐free, non‐fasting conditions yields similar levels of CS‐induced freezing in both groups (Figure 2F, t_(14)_ = 0.42, *P* > 0.05).

**Figure 3:**
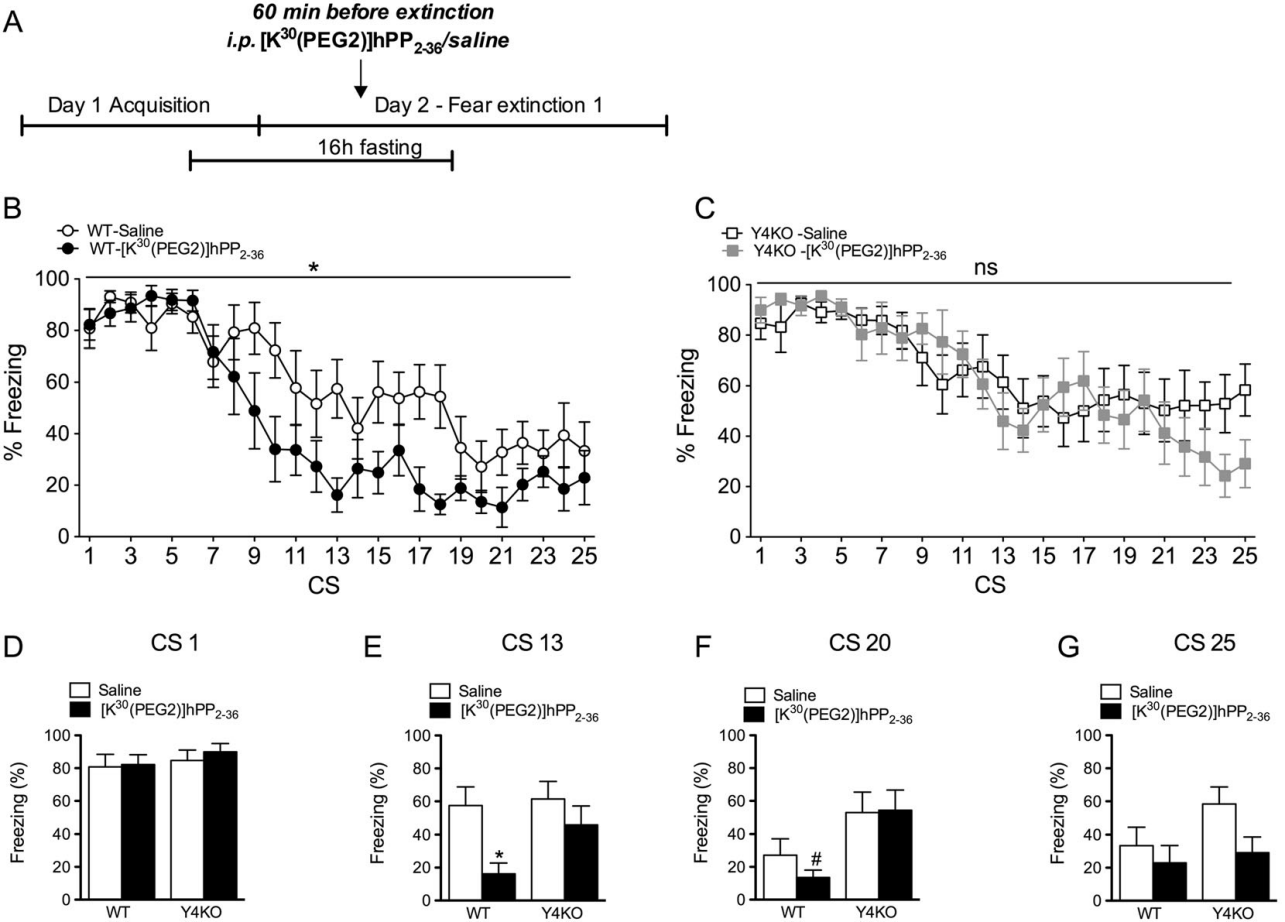
Peripheral injection of [K^30^(PEG2)]hPP_2‐36_ facilitates fear extinction by activation of Y_4_ receptors. (A) Experimental timeline of fear conditioning experiments. (B) Facilitated fear extinction in male WT mice but not in (C) Y4KO mice after peripheral injections of [K^30^(PEG2)]hPP_2‐36_ 60 min before fear extinction. (D) Equal freezing levels of all groups during the first CS, demonstrating equal fear expression, (E) reduced freezing level of [K^30^(PEG2)]hPP_2‐36_‐injected WT mice compared with those of all other experimental groups (CS13). (F) While both WT groups displayed extinction of conditioned fear, Y4KO mice exhibited still high freezing levels (CS20) and (G) extinction of conditioned fear in all four treatment groups at CS 25 (WT: saline: *n* = 10, [K^30^(PEG2)]hPP_2‐36_: *n* = 10; Y4KO: saline: *n* = 10, [K^30^(PEG2)]hPP_2‐36_: *n* = 10; data are expressed as mean ± SEM. *P < 0.05, PP significantly different from saline. #*P* < 0.05, WT significantly different from Y4KO.

Central Y_4_ receptors may be targeted by peripherally released PP; however, also, NPY released in the CNS displays high affinity for Y_4_ receptors (Bard *et al.*, 1995; Gehlert *et al.*, 1996), while PP may act also on Y_6_ receptors (Yulyaningsih *et al.*, 2014). Thus, to investigate if the extinction‐promoting effect is due to peripherally applied PP acting specifically on Y_4_ receptors, we injected [K^30^(PEG2)]hPP_2‐36_ 60 min before extinction in fasted (16 h) Y4KO mice (Figure 3A). In contrast to concomitantly tested WT mice (Figure 3B and E, F_(1/16)_ = 6.18, *P* < 0.05), Y4KO mice did not respond to [K^30^(PEG2)]hPP_2‐36_ injection (Figure 3C–G, F_(1/16)_ = 0.02, *P* > 0.05), indicating the specific activity of [K^30^(PEG2)]hPP_2‐36_ on Y_4_ receptors. Further analysis revealed that WT mice injected with [K^30^(PEG2)]hPP_2‐36_ already displayed reduced freezing at CS13, while no such affect was apparent in Y4KO mice (Figure 3E, F_(1/16)_ = 8.38, *P* < 0.05). Importantly, there was a difference in freezing behaviour at CS20 between WT and Y4KO mice only in the [K^30^(PEG2)]hPP_2‐36_‐treated group (Figure 3F, F_(1/16)_ = 12.02, *P* < 0.05). Although an apparent difference in freezing levels may be indicated during the end of the extinction session (e.g. CS25) also in Y4KO mice, neither the overall course of the extinction session nor a specific testing of CS25 was significantly different. These additional analyses support a drug effect in WT mice, but the absence of an effect in Y4KO mice. Interestingly, and as shown previously (Verma *et al.*, 2016), impaired fear extinction was rescued in Y4KO mice upon fasting, further demonstrating the close relationship of energy homeostasis and emotional processing (Figure 3C and G).

Taken together, we conclude that peripherally injected [K^30^(PEG2)]hPP_2‐36_ specifically promoted cued fear extinction learning by activating Y_4_ receptors in fasted mice.

### Role of PP and Y_4_ receptors in the permanence of extinction memory

**Figure 4:**
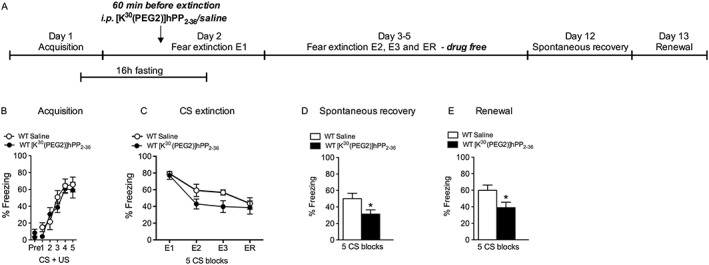
Treatment with [K^30^(PEG2)]hPP_2‐36_ before fear extinction results in long‐term suppression of fear. (A) Experimental timeline of fear conditioning experiments. (B) Male WT mice were divided into two groups with equal fear acquisition, (C) facilitated extinction during three consecutive extinction sessions (E1–E3) in [K^30^(PEG2)]hPP_2‐36_‐treated mice compared with saline‐injected controls and equal freezing levels during extinction recall (ER), but decreased freezing of [K^30^(PEG2)]hPP_2‐36_ group during (D) spontaneous recovery and (E) renewal on days 12 and 13 respectively (saline: *n* = 7, [K^30^(PEG2)]hPP_2‐36_: *n* = 7; data are expressed as mean ± SEM. *P < 0.05, significantly different from saline.

Fear memories are strong and often persist lifelong, whereas extinction memories are labile and transient, resulting in relapse of fear in particular upon stressful situations. To investigate the permanence of extinction memory, we employed two well‐established paradigms, spontaneous recovery and renewal testing. Male WT mice were fasted for 16 h, injected with [K^30^(PEG2)]hPP_2‐36_ or saline and subjected to an extinction training 60 min later on day 2 (Figure 4A). Two additional extinction sessions under drug‐free, non‐fasting conditions were added to yield equal levels of fear expression (Figure 4A). In mice that had received [K^30^(PEG2)]hPP_2‐36_ before extinction training 1, reduced freezing was evident during spontaneous recovery (Figure 4D, t_(12)_ = 2.24, *P* < 0.05) and renewal (Figure 4E, t_(12)_ = 2.28, *P* < 0.05) tests that were performed on days 12 and 13 respectively. These experiments demonstrate that [K^30^(PEG2)]hPP_2‐36_ injection before extinction training creates a long‐lasting extinction memory with permanent suppression of fear.

### Influence of homeostatic balance on the effect of PP on extinction learning

In order to avoid possible interference with endogenously released PP, all previous experiments were performed after a 16 h period of fasting. However, we have previously shown that fasting alone is sufficient to promote extinction learning (Verma *et al.*, 2016). To investigate if short‐term fasting (16 h) controls the responsiveness to PP, we repeated the experiments in non‐fasted mice by injecting [K^30^(PEG2)]hPP_2‐36_ i.p. 60 min before extinction training (Supporting Information Figure S5A). Interestingly, when we performed extinction training in non‐fasted WT mice, peripheral injection of [K^30^(PEG2)]hPP_2‐36_ did not facilitate extinction learning anymore (Figure S5C and D, F_(1/10)_ = 0.30, *P* > 0.05), suggesting that food deprivation is essential for the action of [K^30^(PEG2)]hPP_2‐36_ on fear extinction.

### Effect of peripheral injection of [K^30^(PEG2)]hPP_2‐36_ on the acquisition and consolidation of fear memories

Although male Y4KO mice did not display any alterations in acquisition, consolidation or recall/expression of fear, a possible Y_4_ receptor‐mediated effect may have been masked here by developmental adaptations. Thus, it is important to investigate the acute effects of Y_4_ receptor stimulation during fear acquisition and consolidation in WT mice. To characterize the effect of PP and Y_4_ receptors in fear acquisition and consolidation we injected i.p. [K^30^(PEG2)]hPP_2‐36_ before or after fear acquisition respectively (Supporting Information Figures S6 and S7). Injection of the long‐acting Y_4_ receptor agonist 60 min before fear acquisition did not alter context fear (Figure S6C and D, F_(1/9)_ = 0.70, *P* > 0.05) or CS‐induced fear (t_(8)_ = 0.51, *P* > 0.05) tested 24 and 48 h later respectively. Furthermore, injection of [K^30^(PEG2)]hPP_2‐36_ immediately after fear acquisition, a schedule to test for fear consolidation, did also not change context fear (Figure S7C and D, F_(1/9)_ = 1.35, *P* > 0.05) or CS‐induced freezing (t_(8)_ = 0.45, *P* > 0.05). Collectively, these data suggest that PP does not interfere with the acquisition and consolidation of conditioned fear but rather displays a specific, feeding‐dependent effect on the acquisition of fear extinction.

### Activation of CNS neurons by peripherally injected [K^30^(PEG2)]hPP_2‐36_ during fear extinction

**Figure 5:**
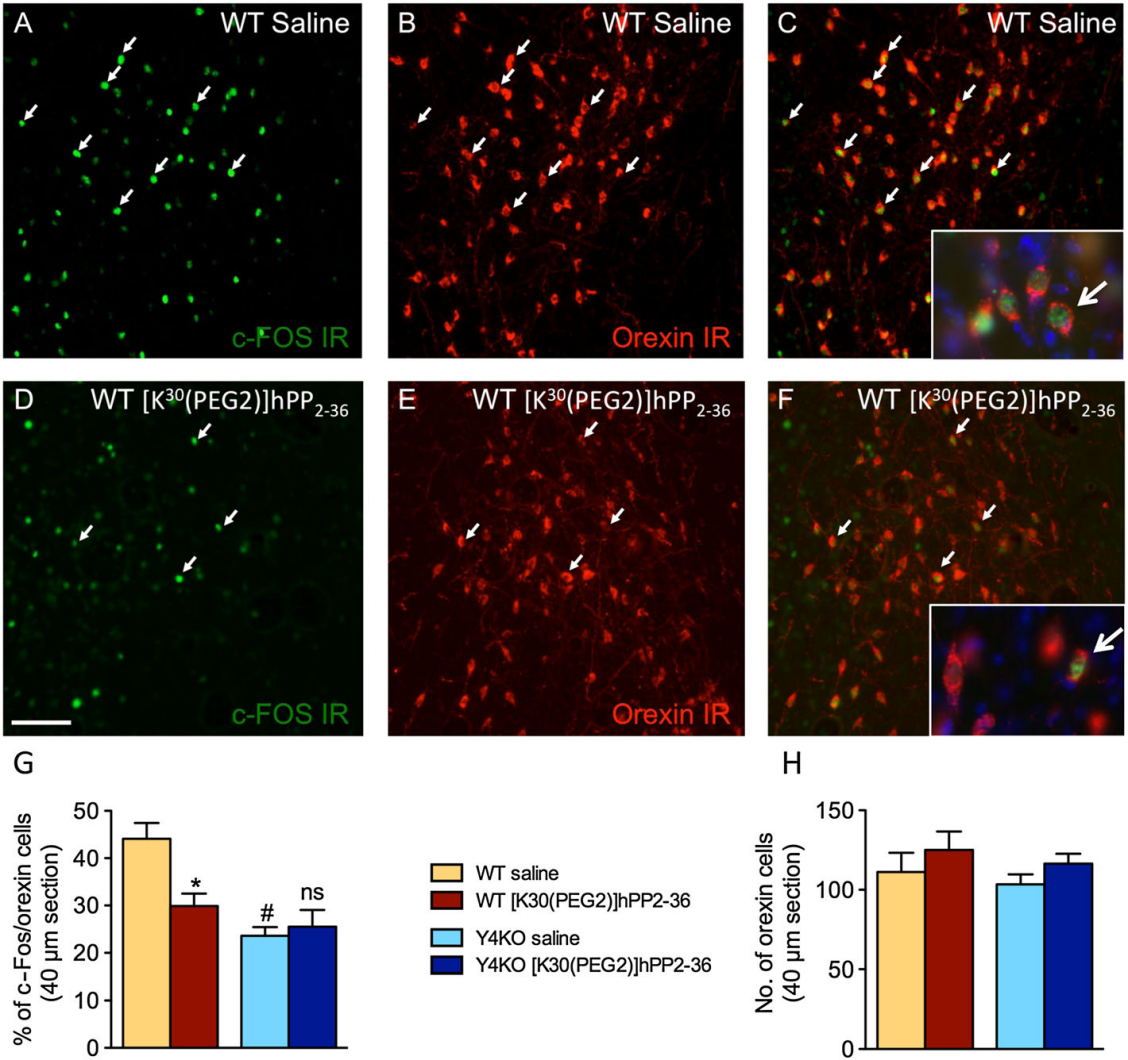
Peripheral injection of [K^30^(PEG2)]hPP_2‐36_ before fear extinction specifically reduces the activation of orexin neurons in the lateral hypothalamus. Male WT and Y4KO mice were fasted before and during fear extinction (16 h) and injected with [K^30^(PEG2)]hPP_2‐36_ 60 min before fear extinction; all mice were perfused 90 min after the end of extinction training. Representative images of immunohistochemistry for c‐Fos, orexin and overlay for (A–C) saline‐injected and (D–F) [K^30^(PEG2)]hPP_2‐36_‐injected WT mice. Arrows point to examples of dual‐labelled neurons, higher magnification in A and F with Hoechst staining for labelling nuclei; scale bar 100 μm. Histograms depicting quantification of (G) percentage of cFos/orexin double‐labelled neurons and (H) number of orexin neurons in the lateral hypothalamus of WT and Y4KO mice (WT: saline: *n* = 7, [K^30^(PEG2)]hPP_2‐36_: *n* = 7; Y4KO: saline: *n* = 7, [K^30^(PEG2)]hPP_2‐36_: *n* = 7; data are expressed as mean ± SEM. *P < 0.05, significantly different from saline. #*P* < 0.05, WT significantly different from Y4KO.

PP released from the pancreas acts on Y_4_ receptors in the periphery but may also target the CNS (Dumont *et al.*, 2007; Tasan *et al.*, 2009). To identify brain areas that are differentially activated by PP during fear extinction learning, WT and Y4KO mice were fasted for 16 h, injected i.p. with [K^30^(PEG2)]hPP_2‐36_ and subjected to fear extinction training. For immediate early gene mapping, brains were removed 90 min after behavioural testing. As shown in Figure 5, peripheral injection of [K^30^(PEG2)]hPP_2‐36_ specifically reduced the percentage of cFos‐positive, orexin neurons in the lateral hypothalamus (Figure 5G) in WT but not in Y4KO mice. These data indicate that fasting and fear exposure result in high arousal during extinction learning and that peripherally injected PP may promote fear extinction in part by reducing stress levels through inhibition of orexin neurons.

## Discussion and conclusions

The important function of neuropeptides in regulating fear‐related and extinction‐related processes is becoming increasingly evident (Bowers *et al.*, 2012; Singewald *et al.*, 2015). However, while most data concern the activity of central neuromodulators, much less is known about the interaction of the gut–brain axis in emotional–affective behaviour (Cryan and Dinan, 2012; Holzer and Farzi, 2014). Here, we demonstrated that peripherally released PP is crucially and specifically involved in the extinction of cued fear probably by acting on central Y_4_ receptors. In particular, we demonstrated that peripheral injection of the Y_4_ receptor agonist [K^30^(PEG2)]hPP_2‐36_ significantly promoted extinction learning in fasted, but not in fed, mice and results in a lasting suppression of fear. The absence of an effect in Y4KO mice suggests that it is mediated by activation of Y_4_ receptors. Furthermore, peripheral application of [K^30^(PEG2)]hPP_2‐36_ before fear extinction training resulted in reduced activation of orexin neurons in the lateral hypothalamus, providing a possible interaction point of central and peripheral peptidergic systems during fear processing. While the percentage of activated cFos/orexin neurons in Y4KO mice did not respond to peripheral injection of [K^30^(PEG2)]hPP_2‐36_, their activation was generally reduced compared with that in WT mice. Multiple changes may occur during embryonic and also postnatal development that may account for this reduced activation upon genetic deletion of Y_4_ receptors. Such a reduced activity of orexin neurons may be even more pronounced under non‐fasted conditions and could in part explain the impaired fear extinction of Y4KO mice.

While Y_4_ receptors are expressed in discrete regions of the brain, such as specific nuclei of the hypothalamus and brain stem, their main ligand, PP, is not expressed in the CNS (Pieribone *et al.*, 1992) but synthesized in endocrine F cells of pancreatic islets (Holzer *et al.*, 2012). PP is released postprandially via the vagus nerve to act locally in the gut, but it is also released into the circulation activating Y_4_ receptors in brain regions that are open to the blood–brain barrier (Dumont *et al.*, 2007).

We have demonstrated recently that peripherally applied hPP specifically activates different brain areas in a time‐dependent manner, suggesting direct and indirect activation of limbic circuits (Tasan *et al.*, 2009). In particular, activation of the NTS may be crucial for the central effects of peripherally released PP. The NTS is important for sensing glucose levels and it responds to hypoglycemia by stimulating food intake, inducing release of stress hormones and increasing sympathetic tone (Renner *et al.*, 2010; Rinaman, 2010; Wu *et al.*, 2012). Interestingly, extinction‐promoting effects are known for glucocorticoids and yohimbine, both compounds that increase stress and sympathetic activity (Barrett and Gonzalez‐Lima, 2004; Yang *et al.*, 2006; Holmes and Quirk, 2010; Blundell *et al.*, 2011). Activation of the NTS may thus promote fear extinction by linking the internal homeostatic situation to emotional arousal. Recent evidence has demonstrated that i.p. injection of radioactive‐labelled [^125^I]hPP specifically accumulates in the area postrema and the NTS, suggesting that peripherally released PP directly acts on Y_4_ receptors in these brain areas (Dumont *et al.*, 2007). NTS neurons have also prominent reciprocal projections to limbic brain areas, in particular to hypothalamic nuclei. Importantly, there is a mutual connection between the NTS and the paraventricular nucleus of the hypothalamus, an area responsible for modulation of sympathetic outflow (Affleck *et al.*, 2012). The important role of the hypothalamus in PP and Y_4_ receptor‐mediated effects is further emphasized by a recent study using MRI scans in fasted rats demonstrating the activation of specific hypothalamic nuclei upon i.p. injection of hPP (Hankir *et al.*, 2011). Furthermore, in the arcuate nucleus of the hypothalamus, PP inhibits GABA neurons by a presynaptic attenuation of glutamate release (Acuna‐Goycolea *et al.*, 2005), presenting thus another potential node of interaction with the central stress system.

We have demonstrated that [K^30^(PEG2)]hPP_2‐36_, acting on central Y_4_ receptors, specifically facilitates cued fear extinction while not affecting fear acquisition and fear consolidation. Extinction of conditioned fear is a learning process that leads to a gradual decrease in fear expression and involves plasticity in a distributed neural network, including prefrontal cortex, amygdala and hippocampus (Orsini and Maren, 2012). Interestingly, the NTS, a brain stem nucleus with high abundance of Y_4_ receptors, has also reciprocal connections with different amygdala nuclei and infralimbic cortex (Ricardo and Koh, 1978; Danielsen *et al.*, 1989; Affleck *et al.*, 2012; Garcia‐Medina and Miranda, 2013). Thus, Y_4_ receptors in the NTS may modify signalling in the amygdala and in the infralimbic cortex, thereby specifically facilitating fear extinction.

Furthermore, we found that the extinction‐promoting effect of PP depended on two important prerequisites: first, the presence of Y_4_ receptors and, second, a defined period of food restriction. In Y4KO mice, impaired extinction acquisition was rescued by a 16 h fasting period but was, however, not further enhanced by peripheral application of [K^30^(PEG2)]hPP_2‐36_, indicating that the extinction‐facilitating effect of PP was mediated by Y_4_ receptors. In WT mice, fasting also accelerated extinction learning, and this effect was further enhanced by injection of [K^30^(PEG2)]hPP_2‐36_ in the present study. In contrast, injection of [K^30^(PEG2)]hPP_2‐36_ did not alter fear extinction in fed WT mice. Thus, [K^30^(PEG2)]hPP_2‐36_ facilitates cued fear extinction only in fasted WT mice, but not in fed WT mice or Y4KO mice. Compared with our previous study (Verma *et al.*, 2016), the slightly higher freezing levels in the current experiments were likely to be due to the stress of i.p. injections shortly before extinction training.

However, short‐term fasting may have contributed to improved extinction learning by increasing arousal. This is also reflected by the activation of orexin neurons in the lateral hypothalamus. Orexin neurons are involved in the modulation of arousal, energy balance and reward processing (Sakurai, 2014). Interestingly, peripherally injected [K^30^(PEG2)]hPP_2‐36_ inhibited the activation of orexin neurons but promoted extinction in fasted mice. Experimental evidence indicates that orexin‐expressing neurons express functional Y_4_ receptors (Campbell *et al.*, 2003) and injection of hPP was suggested to inhibit fasting‐induced increases in orexin mRNA (Sainsbury *et al.*, 2010). However, whether peripherally released PP is able to pass the blood–brain barrier and directly activate Y_4_ receptors on orexin neurons or the inhibition of orexin neurons is rather indirect remains to be demonstrated (Dumont *et al.*, 2007). Orexin and in particular the orexin‐1 receptor have been suggested to increase stress and fear‐related behaviours (Furlong *et al.*, 2009; Steiner *et al.*, 2012; Viviani *et al.*, 2015). Thus, the extinction‐promoting effect of the peripherally applied Y_4_ receptor agonist [K^30^(PEG2)]hPP_2‐36_ may be explained by reducing the orexin‐induced high fear/stress levels evident during extinction learning. More direct, recent data, favouring the hypothesis that PP and Y_4_ receptors mediate the facilitation of extinction by inhibiting orexin neurons, come from the work of Flores *et al*. (2014). The authors demonstrated that blockade of orexin‐1 receptors specifically promotes the consolidation of extinction memory (Flores *et al.*, 2014).

We demonstrated previously that fasting promotes fear extinction in WT mice and can even rescue impaired fear extinction in Y4KO mice (Verma *et al.*, 2016). Many different brain circuits are activated upon acute fasting, in particular during concomitant fear exposure; however, only some of these activity changes may promote fear extinction. For instance, increased attention is necessary for successful extinction learning; however, an overly active arousal system may inhibit extinction consolidation. Thus, in fasted mice, PP may suppress an overstimulated arousal system bringing it back to an efficient level, but this mechanism may be ineffective in fed mice.

Importantly, also, NPY is crucially involved in both food intake (Loh *et al.*, 2015) and modulation of emotional affective behaviour, including fear conditioning and extinction (Tasan *et al.*, 2016). Thus, the NPY system may be at a central node for adapting emotional responses to the present homeostatic situation. It is further conceivable that PP released in the periphery will interact with the central NPY system to achieve this integration of feeding behaviour and emotional processing in the CNS.

A crucial finding is that the suppression of fear behaviour by [K^30^(PEG2)]hPP_2‐36_ treatment before fear extinction is long‐lasting and independent of context as demonstrated by the reduced freezing levels during spontaneous recovery and renewal. Interestingly, freezing behaviour in [K^30^(PEG2)]hPP_2‐36_‐treated mice was even lower than during extinction recall. This suggests that application of [K^30^(PEG2)]hPP_2‐36_ not only promoted extinction learning but also strengthened consolidation and permanence of an acquired extinction memory, an effect that may persist even after washout of the drug. Further experiments are needed to elucidate this effect in more detail.

Fear extinction is considered as neurobiological basis for exposure therapy in humans. However, a major drawback is currently that extinction memory is rather labile resulting in frequent re‐emergence of fear. Thus, a supportive pharmacological intervention that can stabilize an acquired extinction memory during psychotherapy is of high relevance.

Importantly, we highlight here a close interaction of two life‐sustaining circuits, hunger and fear, and we present a peptide that is involved in both feeding and fear. Thus, a thorough analysis of critical mediators of the gut–brain axis may yield further promising drug targets for treating fear and anxiety‐related disorders.

## Author contributions

D.V. and B.H. performed the research; D.V. and R.O.T. analysed the data; H.H. contributed the knockout mice for the study; A.B.S., V.T. and K.B.S. synthesized the Y_4_ receptor agonist [K^30^(PEG2)]hPP_2‐36_ and performed the *in vitro* characterization; R.O.T. designed the research study and R.O.T. and G.S. wrote the paper.

## Conflict of interest

The authors declare no conflicts of interest.

## Declaration of transparency and scientific rigour

This Declaration acknowledges that this paper adheres to the principles for transparent reporting and scientific rigour of preclinical research recommended by funding agencies, publishers and other organizations engaged with supporting research.

## Supporting information


**Figure S1** RP‐HPL chromatogram (A) and MALDI‐TOF mass spectrum (B) of [K^30^(PEG2)]hPP. RP‐HPLC was performed on a Jupiter 4u Proteo 90 Å (Phenomenex) applying a gradient of 20 to 60% of eluent B (0.08% TFA in acetonitrile) in eluent A (0.1% TFA in water) over 40 min.
**Figure S2** Inositol phosphate turnover assay of the activity of [K^30^(PEG2)]hPP_2‐36_ at all human Y receptors. Inositol phosphate accumulation was measured by stimulating COS7 cells stably transfected with the respective Y receptor subtype and a chimeric G_i/q_‐protein with different peptide concentrations. Experiments were performed in duplicate, at least twice. Data points shown are means ± SEM. Curves were generated by nonlinear regression using GraphPad Prism 5.0.
**Figure S3** Proteolytic stability of [K^30^(PEG2)]hPP_2‐36_ in human blood plasma. Peptide was incubated in human blood plasma at 37°C for the indicated periods. The degradation assay was carried out in two independent experiments (n = 2) and results are presented as means ± SEM.
**Figure S4** Activity measurements over 24 h after i.p. injection of a Y_4_ receptor agonist or saline. Motor activity in a novel cage was unchanged after i.p. injection of the Y_4_ receptor agonist [K^30^(PEG2)]hPP_2‐36_ (saline: *n* = 7, [K^30^(PEG2)]hPP_2‐36_: *n* = 8; data are expressed as mean ± SEM).
**Figure S5** Peripheral [K^30^(PEG2)]hPP_2‐36_ injection does not affect fear extinction in non‐fasted mice. (A) Experimental timeline of fear conditioning and extinction experiments, (B) after fear acquisition mice were divided into two equal groups, (C) fear extinction and (D) extinction recall, is similar between non‐fasted [K^30^(PEG2)]hPP_2‐36_‐injected WT mice and saline‐injected controls (saline: *n* = 6, [K^30^(PEG2)]hPP_2‐36_: *n* = 6; data are expressed as mean ± SEM).
**Figure S6** Peripheral injection of the Y_4_ receptor agonist [K^30^(PEG2)]hPP_2‐36_ does not influence acquisition of conditioned fear. (A) Experimental set‐up with 16 h of fasting before and during fear acquisition, (B) no change in fear acquisition, (C) context fear and (D) CS‐induced fear expression after peripheral injection of [K^30^(PEG2)]hPP_2‐36_ 60 min before fear acquisition compared with saline‐injected controls (saline: *n* = 6, [K^30^(PEG2)]hPP_2‐36_: *n* = 6; data are expressed as mean ± SEM).
**Figure S7** The Y_4_ receptor agonist [K^30^(PEG2)]hPP_2‐36_ did not affect the consolidation of conditioned fear. (A) Experimental procedure of fear conditioning experiments. (B) Following fear acquisition, mice were divided into two groups that were injected with [K^30^(PEG2)]hPP_2‐36_ or saline, (C) no change in context fear extinction and (D) CS‐induced freezing of [K^30^(PEG2)]hPP_2‐36_ injected mice compared to saline injected controls (saline: *n* = 6, [K^30^(PEG2)]hPP_2‐36_ n=6; data are expressed as mean ± SEM).

Supporting info item
