# Supplementary material for: Pancreatic polypeptide and its central Y4 receptors are essential for cued fear extinction and permanent suppression of fear
Source: Br J Pharmacol. 2016 May 19;173(12):1925–38. doi: 10.1111/bph.13456 (PMC4882497; doi:10.1111/bph.13456)
Supplement: Supplementary file 1 — Supporting info item [file BPH-173-1925-s001.docx]

**Supplementary Figures**


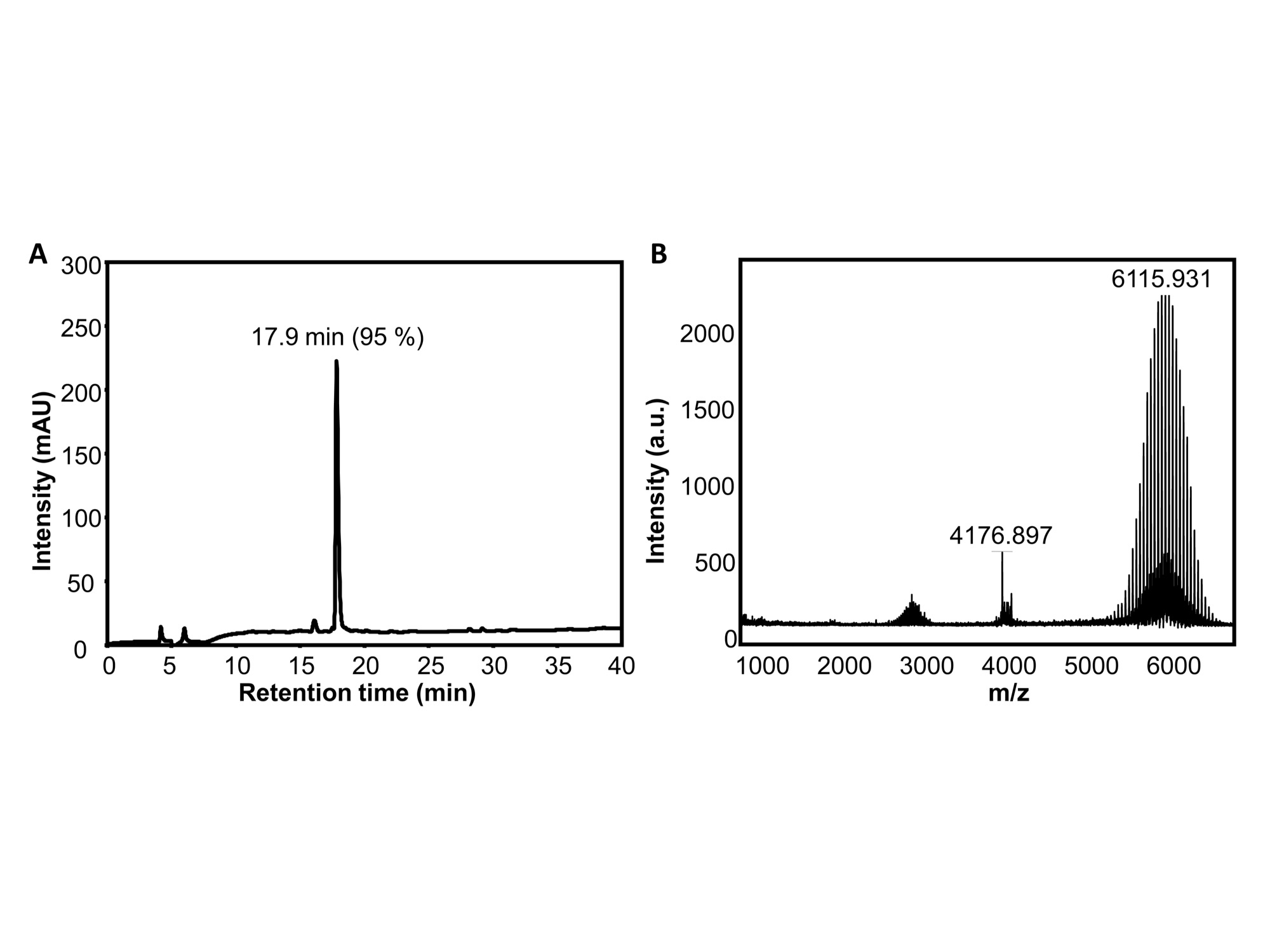


**Supplementary Figure 1.** RP-HPL chromatogram (A) and MALDI-TOF mass spectrum (B) of [K^30^(PEG2)]hPP. RP-HPLC was performed on a Jupiter 4u Proteo 90 Å (Phenomenex) applying a gradient of 20 to 60 % of eluent B (0.08% TFA in acetonitrile) in eluent A (0.1% TFA in water) over 40 min.


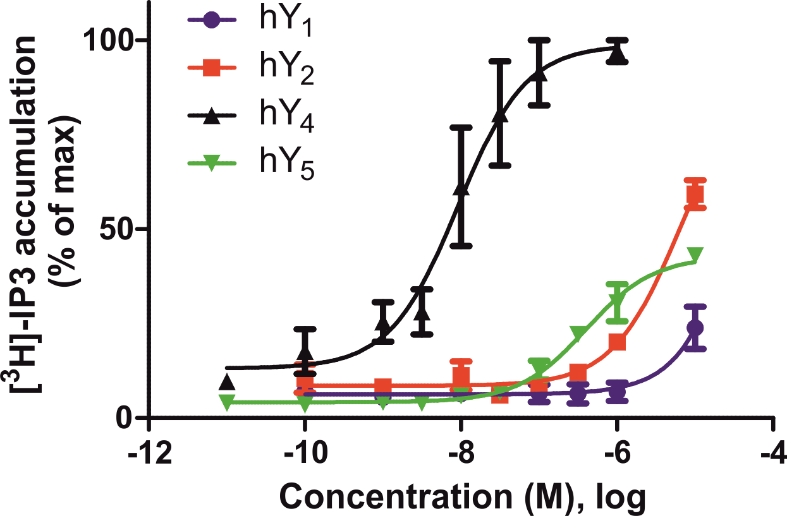


**Supplementary Figure 2.** Inositol phosphate turnover assay of activity of [K^30^(PEG2)]hPP_2-36_ at all human Y receptors. Inositol phosphate accumulation was measured by stimulating COS7 cells stably transfected with the respective Y receptor subtype and a chimeric G_i/q_-protein with different peptide concentrations. Experiments were performed in duplicate, at least twice. Data points shown are mean ± SEM. Curves were generated by nonlinear regression using GraphPad Prism 5.0.


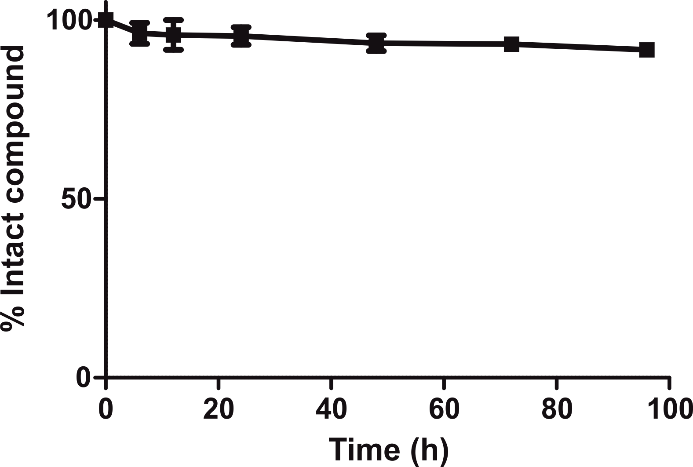


**Supplementary Figure 3.** Proteolytic stability of [K^30^(PEG2)]hPP_2-36_ in human blood plasma. Peptide was incubated in human blood plasma at 37°C for the indicated periods. The degradation assay was carried out in two independent experiments (n = 2) and results are presented as means ± SEM.

**
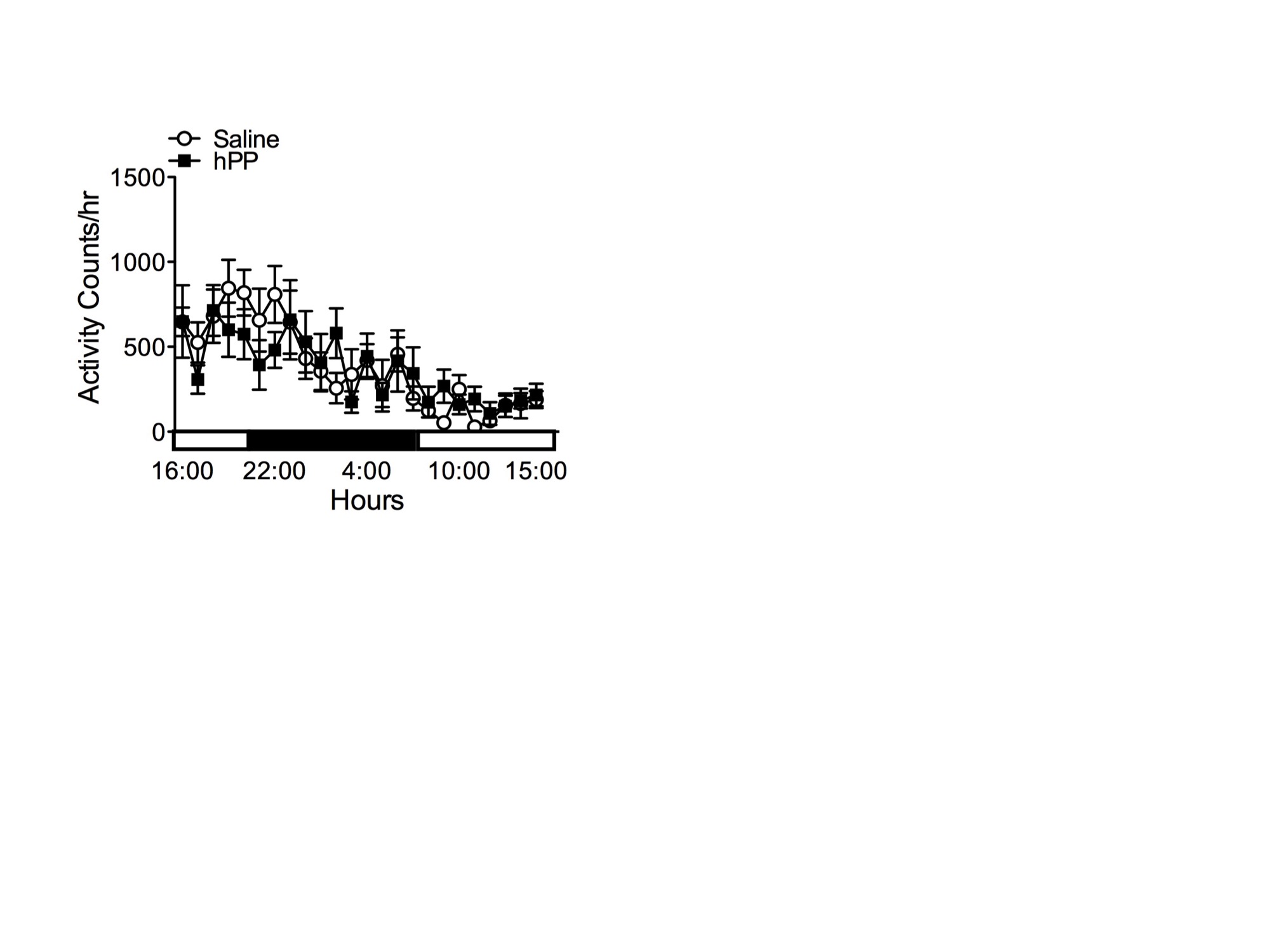
**

**Supplementary Figure 4.** Activity measurements over 24h after i.p. injection of a Y4 receptor agonist or saline. Motor activity in a novel cage was unchanged after i.p. injection of the Y4 receptor agonist [K^30^(PEG2)]hPP_2-36_ (saline: n=7, [K^30^(PEG2)]hPP_2-36_: n=8; data are expressed as mean ± SEM).


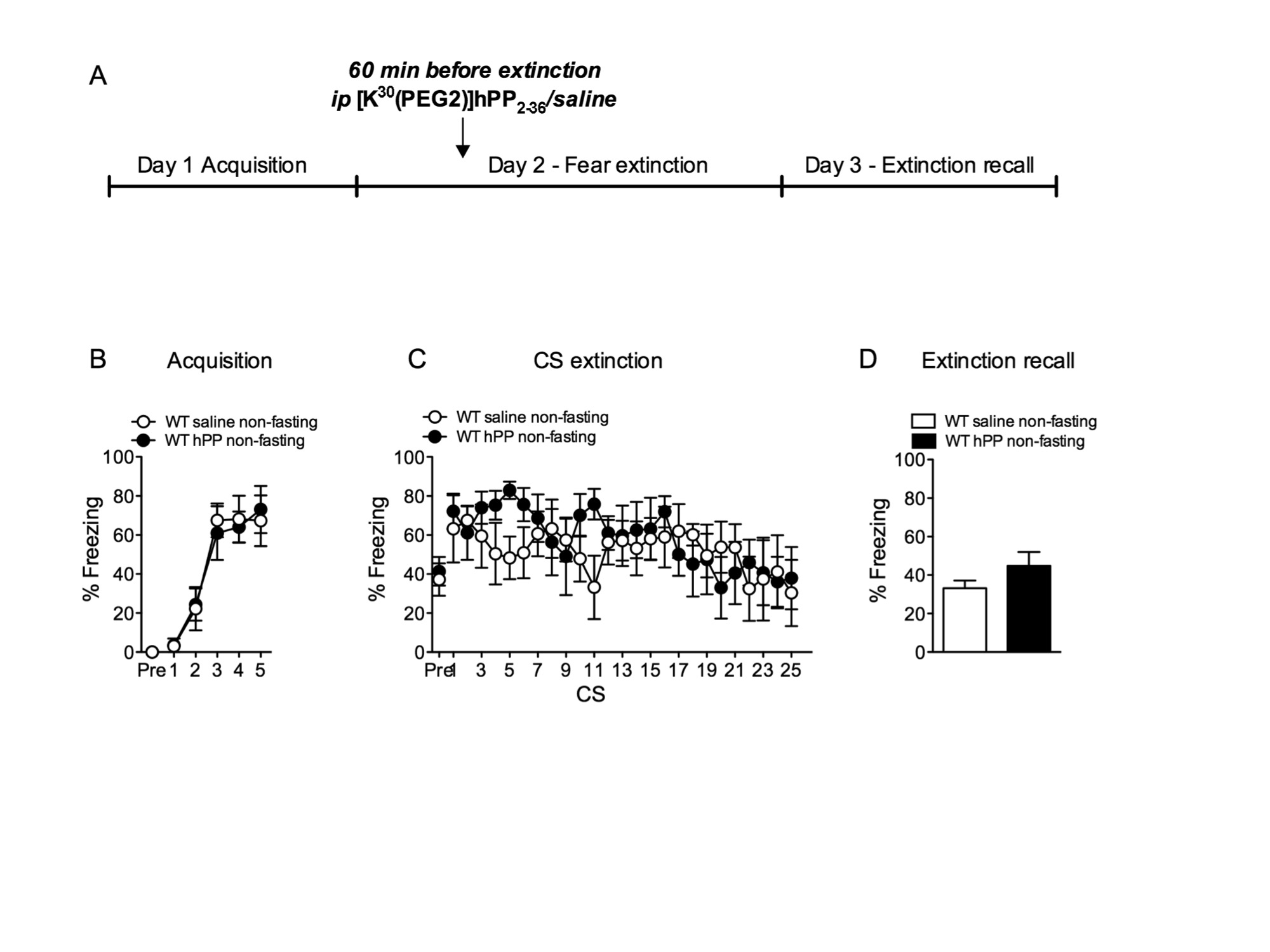


**Supplementary Figure 5.** Peripheral [K^30^(PEG2)]hPP_2-36_ injection does not affect fear extinction in non-fasted mice. (A) Experimental timeline of fear conditioning and extinction experiments, (B) after fear acquisition mice were divided into two equal groups, (C) fear extinction and (D) extinction recall is similar between non-fasted [K^30^(PEG2)]hPP_2-36_ injected WT mice and saline injected controls (saline: n=6, [K^30^(PEG2)]hPP_2-36_: n=6; data are expressed as mean ± SEM).


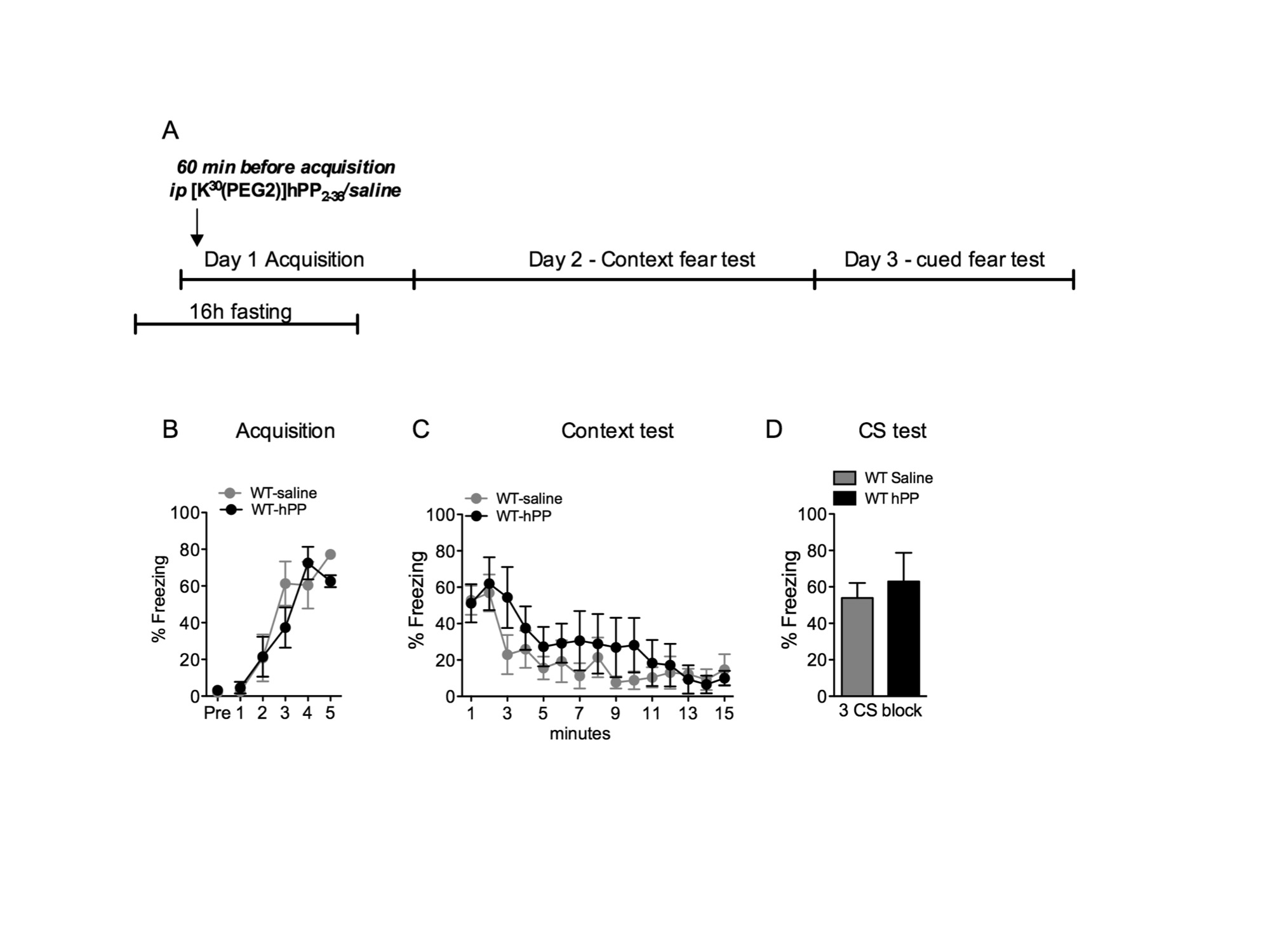


**Supplementary Figure 6.** Peripheral injection of [K^30^(PEG2)]hPP_2-36_ did not influence acquisition of conditioned fear. (A) Experimental setup with 16 hours of fasting before and during fear acquisition, (B) no change in fear acquisition, (C) context fear and (D) CS-induced fear expression after peripheral injection of [K^30^(PEG2)]hPP_2-36_ 60 min before fear acquisition compared to saline injected controls (saline: n=6, [K^30^(PEG2)]hPP_2-36_: n=6; data are expressed as mean ± SEM).


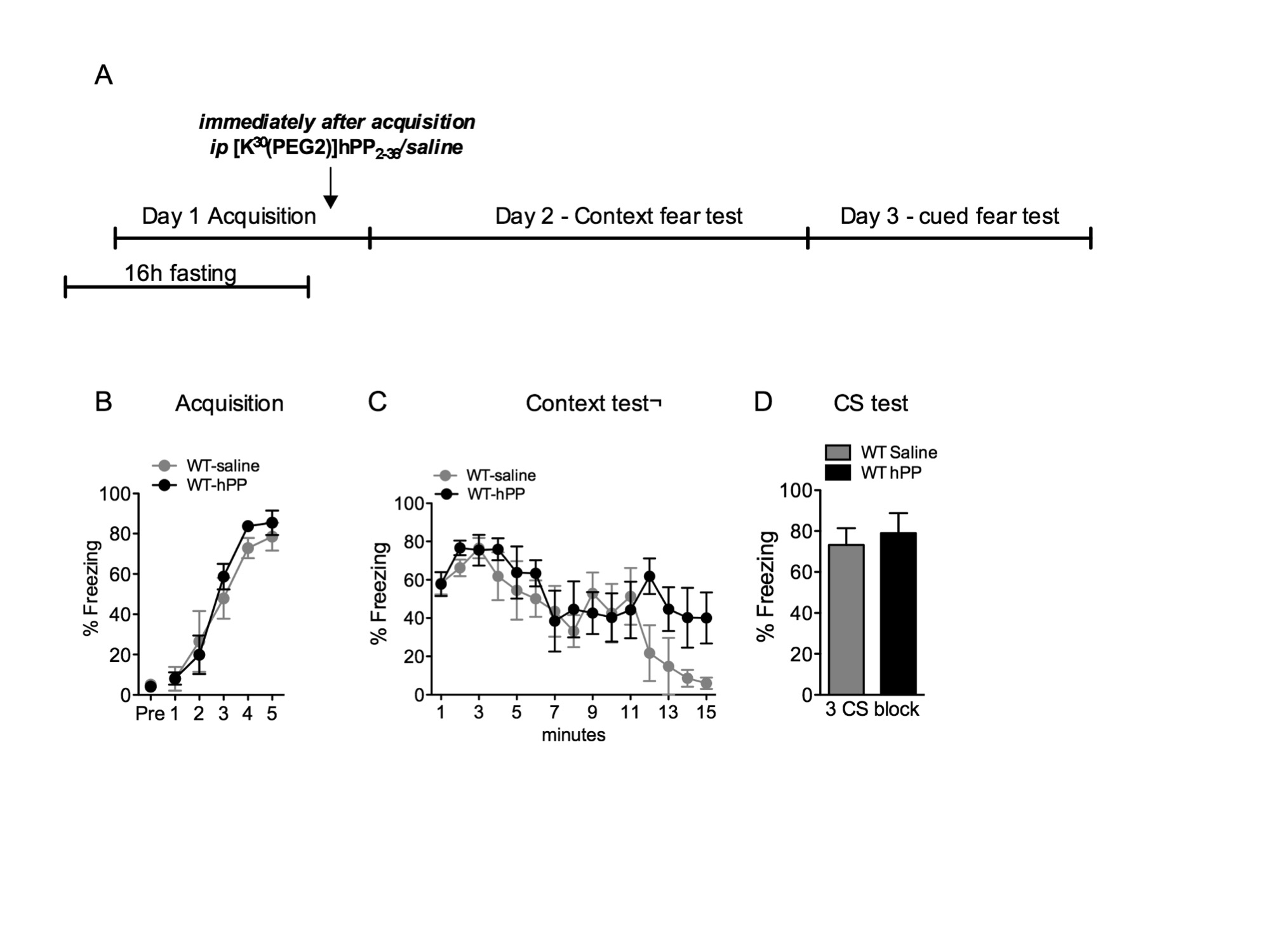


**Supplementary Figure 7.** The Y_4_ receptor agonist [K^30^(PEG2)]hPP_2-36_ does not affect the consolidation of conditioned fear. (A) Experimental procedure of fear conditioning experiments. (B) Following fear acquisition, mice were divided into two groups that were injected with [K^30^(PEG2)]hPP_2-36_ or saline, (C) no change in context fear extinction and (D) CS-induced freezing of [K^30^(PEG2)]hPP_2-36_ injected mice compared to saline injected controls (saline: n=6, [K^30^(PEG2)]hPP_2-36_: n=6; data are expressed as mean ± SEM).
